# Music as a window into real-world communication

**DOI:** 10.3389/fpsyg.2023.1012839

**Published:** 2023-07-11

**Authors:** Sarah C. Izen, Riesa Y. Cassano-Coleman, Elise A. Piazza

**Affiliations:** Department of Brain and Cognitive Sciences, University of Rochester, Rochester, NY, United States

**Keywords:** music, communication, naturalistic, neuroimaging, joint action, social

## Abstract

Communication has been studied extensively in the context of speech and language. While speech is tremendously effective at transferring ideas between people, music is another communicative mode that has a unique power to bring people together and transmit a rich tapestry of emotions, through joint music-making and listening in a variety of everyday contexts. Research has begun to examine the behavioral and neural correlates of the joint action required for successful musical interactions, but it has yet to fully account for the rich, dynamic, multimodal nature of musical communication. We review the current literature in this area and propose that naturalistic musical paradigms will open up new ways to study communication more broadly.

## Introduction

When two people communicate, they exchange information, ideas, or feelings, typically through dialogue. While the vast majority of communication research has focused on speech and language, another universal and emotionally powerful medium of human communication is music. Music allows parents to bond with babies, friends to form lifelong memories while singing together on a car trip, and performers to feel a rush of connection with other players and audience members.

Music’s evolutionary origins are rooted in communicative functions (Savage et al., 2021), with one example being the use of African talking drums to communicate messages across villages, including emotional information (Carrington, 1971; Ong, 1977). Music and verbal communication share many common features. For example, music relies primarily on acoustic signaling but is highly multisensory, involving social cues such as gesture and facial expression. Music and language rely on overlapping neural substrates (see Peretz et al., 2015 for a review), both recruiting a hierarchy progressing from primary auditory cortex to higher-order brain regions that process successively longer syntactic units (Lerner et al., 2011; Farbood et al., 2015). This hierarchical structure has also been observed in auditory samples taken from conversations, musical interactions, and even in communications between animals such as killer whales (Kello et al., 2017). Many studies have focused on the ontological and neural overlap and mutual influence between music and language (see Peretz et al., 2015 for a review). However, music connects people in ways that transcend language, thus providing a unique lens into certain aspects of communication largely overlooked or less relevant in language research. Perhaps more than almost any other medium, music communicates a rich tapestry of emotional content, another type of communicable information (Parkinson, 2011), both within a musical group and to the audience. As a collective art form, music plays an important role in establishing shared cultural identity through ritual (e.g., at weddings, funerals, festivals), in which its powers of emotional communication play a large role. In film and television, music adds emotional depth to critical plot points, and it can unlock autobiographical memories in patients with dementia (El Haj et al., 2012a,b). In essence, it provides the social–emotional fabric of many people’s lives, both in the moment and through the ongoing construction of our personal and collective social narratives. While music’s activation of specific emotional processing regions in individual listeners has been fairly extensively studied (Koelsch, 2014), there is little work examining how multiple performers’ brains jointly represent the moment-to-moment dynamics of musical emotion and how these representations relate to listeners’ ongoing experiences.

**Figure 1:**
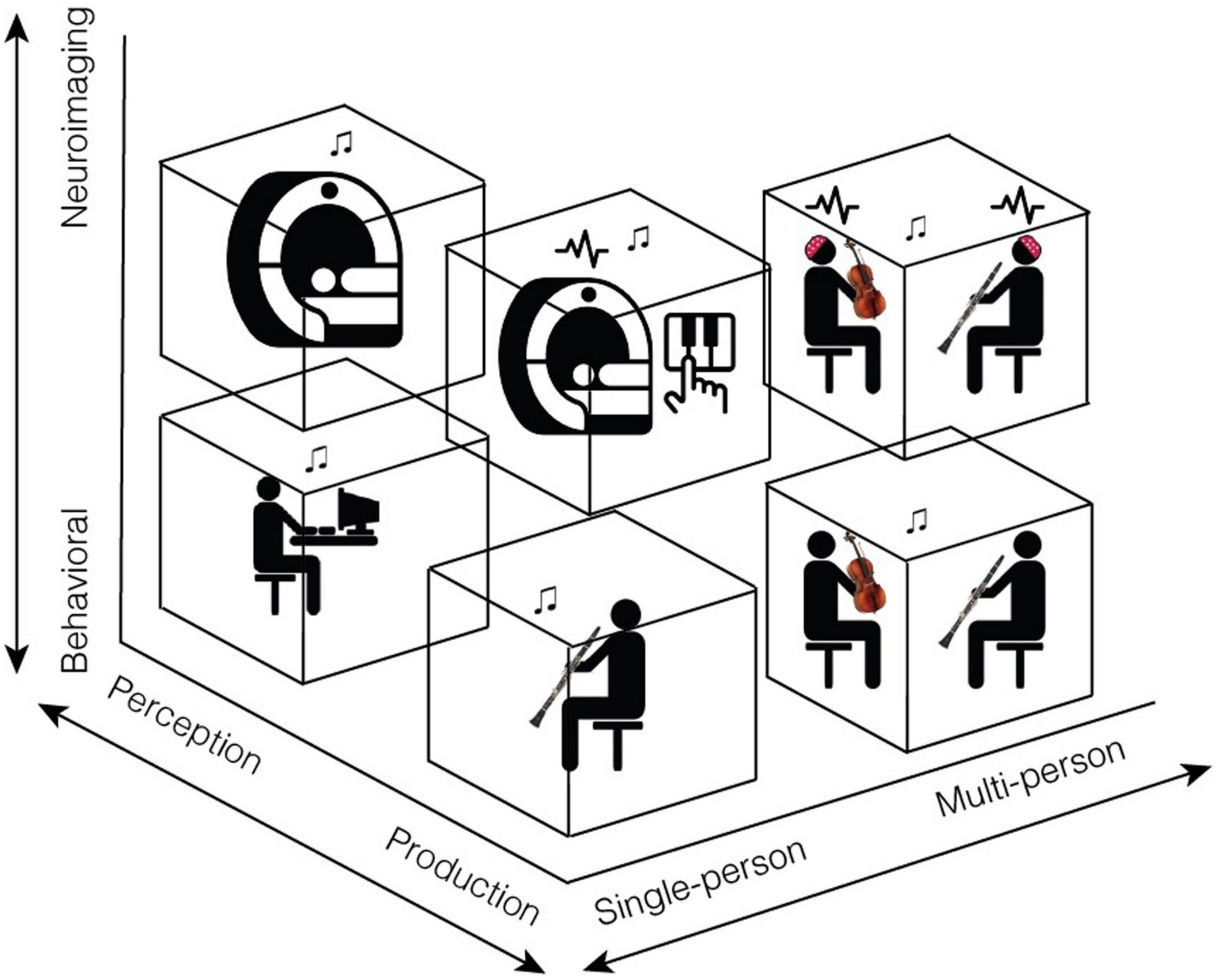
Our proposed framework for future studies of communication includes three methodological axes: behavioral to neuroimaging, perception to production, and single- to multi-person. Single-person behavioral studies of perception, which are already quite common, include psychophysical experiments or survey methods. Single-person neuroimaging studies of perception typically include EEG or fMRI experiments in which participants listen to music. Studying music production in a single person behaviorally involves examining the way a single musician communicates information as they play, whether by adjustments in tempo, dynamics, body sway, or other features. Finally, multi-person studies are essential for capturing the real- world dynamics of everyday musical communication. For example, we propose neuroimaging experiments in which a person’s brain responses are measured while they interact with a live partner or are made to believe they are. Such studies could examine the way musicians jointly attend to one another and adjust to musical partners, how they communicate emotion both to each other and to an audience, and how representations in brain areas involved in these joint actions dynamically change across the interaction. This figure contains icons from thenounproject.com: Activity by mikicon, Mri by Flowicon, Music Note by Nico Ilk, and Piano by Adrien Coquet.

Another key component of communication between performers takes the form of joint action. Although conversational speech does involve turn-taking and mirroring of body movements (e.g., gestures, head nods), the degree of interpersonal bodily coordination involved in a musical interaction is typically much more precisely temporally aligned, can have a large impact on the interpretation of a piece, and is often required for the interaction to run smoothly. For exampleed fo, musicians must breathe together to align note onsets, and coordinate head, arm and even leg movements to maintain a steady tempo. If this bodily system of the ensemble becomes misaligned, it can have disastrous effects on the musical outcome, potentially requiring the players to start over. Beyond large-scale, visible bodily coordination, players also must precisely coordinate intonation by making micro-adjustments to embouchure and strings, which involves listening and matching at multiple levels (see Supplementary Video Figure 1). Broadly, communication is a requirement for coordination, which is particularly relevant for joint music-making among all types of human interactions. Although incidental synchrony could theoretically occur in the absence of communication (e.g., if two people played at the same time by individually following the beat of a conductor without seeing or hearing each other), this would not represent musical coordination or communication between players. Lacking the in-the-moment, interpersonal adaptation that makes every performance unique, this would almost certainly yield a less naturalistic, less cohesive, less beautiful result.

Traditionally, most studies of human communication have focused on language and have investigated processing and learning of highly controlled linguistic units. However, modern approaches have begun to feature more naturalistic stimuli, such as stories (Huth et al., 2016), movies (Chen et al., 2017), and lectures (Meshulam et al., 2021), and real-world tasks, such as live interactions between multiple interlocutors using hyperscanning paradigms (Dikker et al., 2017; Dai et al., 2018). In this review, we propose applying novel and naturalistic approaches to characterizing the myriad of complex cognitive, social, and emotional processes that enable playing music with others. We highlight new ways of measuring crucial but largely overlooked aspects of everyday musical communication: the dynamic exchange of acoustic and gestural signals, the interpersonal coordination that supports rhythmic and harmonic alignment, and the uniquely rich emotional exchange between performers (and between performers and listeners).

Another unique feature of music that enriches its contribution to communication research is the diversity of musical interactions that exist in the world. Virtually all humans are experts at using language to communicate, but the depth and type of musical engagement varies drastically across individuals and cultures, enabling the investigation of musical communication across a range of abilities, modes, genres, and contexts (e.g., amateur vs. professional, instrumental vs. vocal, classical vs. rock, theater vs. church vs. karaoke bar). Finally, music’s potential as an educational and therapeutic tool has only begun to be explored, and we highlight the ways in which research on naturalistic musical communication can lead to more powerful interventions in classroom and clinical settings.

## How has real-world musical communication been studied and which questions remain unanswered?

Joint music-making is unquestionably a highly social activity (see Mehr et al., 2021; Savage et al., 2021 for a debate on its evolutionary origins). Over the course of a musical interaction, musicians must jointly attend to ensure that they are aligning to one another (rhythmically, harmonically, affectively) and flexibly reacting to moment-to-moment changes enacted by their musical partner(s), resulting in mutual entrainment on several levels. Most previous studies examining the behavioral and neural underpinnings of communication have largely ignored these dynamic features, which are critical components of everyday interactions (see Redcay and Schilbach, 2019).

Some researchers have begun to investigate behavioral coordination between multiple musicians (see Volpe et al., 2016 for a review). The string quartet is an excellent model for the study of small group dynamics and communication; because there is no conductor leading them, the musicians must jointly attend to one another in order to play together. For example, musicians must expertly direct attention simultaneously to the written music and, peripherally, to the other players to maintain synchrony. Quartets have been used as a proxy for intense work groups, and the success of a quartet predicts measures of its conflict management and group effectiveness (Murnighan and Conlon, 1991). Members of string quartets become empathetically attuned to one another during a performance (Seddon and Biasutti, 2009), and body sway reflects joint emotional expression (Chang et al., 2019). Players identify and correct for timing asynchronies, and these adaptive strategies often vary based on each quartet’s unique dynamics (Wing et al., 2014). Joint action also requires separately tracking and differentiating one’s own actions versus a partner’s over time, which facilitates interpersonal coordination (Loehr et al., 2013; Liebermann-Jordanidis et al., 2021) (See Supplementary Video Figure 1 for a demonstration of the dynamic joint actions required to support successful musical communication in a similar small group, a woodwind quintet).

To answer questions about musical communication at the neural level, researchers have used a variety of neuroimaging paradigms. In single-brain approaches, researchers collect data from only one participant’s brain, while that person communicates with an in-person or virtual partner, or is made to believe they are (Donnay et al., 2014). In dual-brain (hyperscanning) approaches, researchers collect data simultaneously from two participants’ brains during a social interaction (see Czeszumski et al., 2020 and Montague et al., 2002 for reviews). Both types of paradigms can make use of electroencephalography (EEG), functional magnetic resonance imaging (fMRI), electrocorticography (ECoG), and/or functional near-infrared spectroscopy (fNIRS) to answer different questions. EEG has millisecond-level temporal resolution but poor spatial resolution and is best suited to answer questions about the brain’s response to a particular event in time (e.g., a listener’s response to a musician’s change in tempo). By contrast, fMRI has good spatial resolution but poor temporal resolution, and is best suited to answer questions regarding the patterns of activation in certain brain regions during a particular task or listening condition. ECoG offers high spatial and temporal resolution and enables good coverage of the temporal lobe, but the practical constraints of working with surgical patients limit the feasibility of naturalistic, interactive musical tasks to some degree. Lastly, fNIRS has higher spatial resolution than EEG and comparable temporal resolution to fMRI but is relatively cost-effective and minimally susceptible to motion artifacts, making it well-suited for naturalistic hyperscanning paradigms (Czeszumski et al., 2020).

Studying live interactions between multiple musicians at once enables the characterization of interpersonal dynamics that underlie everyday music-making, such as mutual entrainment and joint improvisation. Entrainment can involve alignment (synchronization) to an external beat maintained by a metronome or conductor, whereas mutual entrainment additionally involves moment-to-moment adaptive adjustments between multiple players to maintain a steady meter amidst local changes in tempo. Behavioral studies have shown that two people can jointly entrain to a beat, and that being paired with a musician increases a non-musician’s ability to maintain a steady beat via tapping (Schultz and Palmer, 2019). During joint music-making, humans temporally and affectively entrain to one another (see Phillips-Silver and Keller, 2012 for a review), resulting in synchronous brain activity (Zamm et al., 2021). Using EEG paradigms, between-brain oscillatory couplings both before and during dyadic guitar playing have been linked to interpersonally coordinated actions (Lindenberger et al., 2009; Sänger et al., 2012). Additionally, different patterns of directionality in brain-to-brain synchronization are associated with leader and follower roles during guitar playing (Sänger et al., 2013). Similarly, during joint piano playing, alpha oscillations index participants’ knowledge about their own actions as well as their musical partner’s (Novembre et al., 2016). In an fNIRS study, distinct blood oxygenation patterns in temporo-parietal and somatosensory areas were found to be associated with different violin parts in a duo (Vanzella et al., 2019).

The inferior frontal cortex (IFC) has been implicated in several studies of communicative interaction, both because of its role in language processing and its involvement in the mirror neuron system for joint action (Rizzolatti and Craighero, 2004; Stephens et al., 2010; Jiang et al., 2012). Studies using fNIRS have revealed neural synchronization in the left IFC while two people sang or hummed together (Osaka et al., 2015), and the strength of neural synchronization in bilateral IFC between learners and instructors during interactive song learning predicts behavioral performance (Pan et al., 2018).

While most everyday verbal communication (e.g., dialogue) is improvised, musical communication regularly takes both scripted and improvised forms. The difference between these forms of joint action lies in performers’ reliance on planned versus emergent coordination mechanisms (Goupil and Aucouturier, 2021). During improvisation between musical partners, not only do shared intentions emerge and cause coordination between musicians, but third-party listeners can identify the musicians’ goals (e.g., finding a good ending for oneself or the group as a whole). Hyper-networks based on an interplay of different EEG frequencies are involved in leader/follower roles during guitar improvisation (Müller et al., 2013), and musical improvisation enhances interpersonal coordination, promoting alignment of body movements in a subsequent conversation (Robledo et al., 2021). fMRI research has begun to examine the neural underpinnings of jazz improvisation, finding deactivation of the dorsolateral prefrontal cortex (DLPFC), associated with creative thinking or being in a state of “flow” (Limb and Braun, 2008). During the highly communicative exchange known as “trading fours,” activation in areas related to syntactic processing was observed, suggesting these areas play a domain-general role in communication beyond language (Donnay et al., 2014). However, very few neuroimaging studies have asked pairs or groups of musicians to improvise freely and exchange musical ideas back and forth. Finally, and perhaps counterintuitively, even very large groups improvising together can attain a high level of coordination and joint action during complex tasks without much external structure (Goupil et al., 2020).

The existing hyperscanning literature largely focuses on mirrored synchrony and phase-locking to stimuli or to a communicative partner measured across an entire interaction, but future dual-brain approaches must embrace the moment-to-moment, back-and-forth, nature of music-making, including the way musicians adapt to one another on a number of features and how that process is modulated by expertise. This will require measuring different kinds of interpersonal coupling in fine-grained time bins organized according to constantly changing musical content. For instance, EEG could be used to examine the precise timing of the brain’s response in musician A to a change in tempo, pitch, or rhythm by musician B and the resulting behavioral change. fMRI could be used to measure how a musician’s brain adapts to unexpected musical content (e.g., a non-diatonic note or syncopated rhythm) initiated by a partner outside of the scanner. fNIRS could be used to examine moment-to-moment adaptation to a communicative partner in pairs of musicians, across multiple brain regions at once. EEG could be used simultaneously in many musicians to examine the leader/follower temporal dynamics of orchestral music. Motion capture could be used in conjunction with hyperscanning to assess how the brain represents the dynamics of gesture over time. Finally, while previous studies have used hyperscanning to examine scripted music-making, future studies should directly compare scripted versus improvised musical interactions, as well as joint versus solo improvisation.

## How is emotion communicated during joint music-making?

Music is inherently emotional (Juslin, 2000), enabling it to support important social functions when played or even listened to. Performing in an ensemble has a uniquely powerful bonding effect between players (and between players and audiences), and simply listening to music often occurs in the context of social experiences that have huge emotional implications (e.g., hearing a sweeping movie score and feeling connected to the other people in the theater or listening to a nostalgic song with a friend during a road trip). However, emotion has been largely ignored in more ecologically valid hyperscanning paradigms examining musical communication (Acquadro et al., 2016). Indeed, music and speech overlap to some degree in their ability to communicate emotion, with a shared mechanism responsible for affective processing of musical and vocal stimuli in the auditory cortex (Paquette et al., 2018) and shared acoustic codes (Curtis and Bharucha, 2010; Coutinho and Dibben, 2013; Nordström and Laukka, 2019). Across speech and music, participants’ emotional experiences can be predicted by the same seven features: loudness, tempo/speech rate, melody/prosody contour, spectral centroid, spectral flux, sharpness, and roughness (Coutinho and Dibben, 2013), and the minor third is associated with sadness both in music and in speech prosody (Curtis and Bharucha, 2010).

Music often triggers physiological responses like chills, changes in skin conductance, and changes in heart rate. When participants listened to music that induced chills, areas of the brain associated with reward/motivation, emotion, and arousal were found to be more active (Blood and Zatorre, 2001). Additionally, those who experience chills show higher white matter connectivity between auditory, social, and reward-processing areas (Sachs et al., 2016). Further, feeling moved or touched by music shares common physiological changes with feeling moved or touched by videos of emotional social interactions, like an elephant reuniting with its mother (Schubert et al., 2018; Vuoskoski et al., 2022).

While there is an intuitive notion that music has the ability to communicate emotion even more powerfully than speech, there is minimal direct empirical evidence for this. Developmental research has shown that song is reliably more effective than speech at modulating emotion in infants (Corbeil et al., 2013; Trehub et al., 2016; Cirelli and Trehub, 2020). And in a dual-brain fNIRS study with adults, the right temporo-parietal junction (rTPJ), which has been previously implicated in social and emotional communication, was found to be more strongly activated during joint drumming than conversation (Rojiani et al., 2018). However, the relative efficacy of music versus speech to communicate emotional information is still largely unknown.

Future studies must delineate the multiple ways that musical emotion is communicated during everyday experiences: the emotions a listener experiences as they hear live or recorded music, the emotions a performer experiences as they play with others or receive feedback from listeners, and the way the communicated emotion helps to create a cohesive social unit between people. Such studies could use behavioral methods and questionnaires to assess emotional states before and after music making, ask performers or listeners to continuously indicate (e.g., via button press or slider) when they enter a new emotional state throughout a performance, and/or use multivariate pattern extraction techniques in neuroimaging paradigms to decode the fine-grained patterns of brain responses in emotion-associated areas as these states change over the course of music-making. Further, while research suggests that song has a unique emotional power over speech in infants (Corbeil et al., 2013; Trehub et al., 2016; Cirelli and Trehub, 2020), it remains to be seen if this effect persists into adulthood. Thus, future studies would benefit from directly comparing emotional communication using musical versus matched linguistic stimuli in adults.

## Musical engagement exists on a spectrum: how do individual differences in musical engagement affect musical communication–and everyday communication more broadly?

One major way in which communication via music differs from communication via language is that in many cultures, musical expertise—as well as degree and type of musical engagement—varies widely in a population. Such variability also exists across cultures: in some cultures, joint music-making is a crucial part of everyday life and rituals, and in others, music is less a part of everyday life and is something that is reserved for certain occasions and highly trained experts. How do differences in musical engagement affect musical communication and translate into everyday social communication?

Most people engage with music in some way on a daily basis—overhearing it at the grocery store, actively listening to recordings at home, attending live concerts among other audience members, singing karaoke with friends, or performing in an ensemble. In many of these examples, music acts as a social reinforcer, but it is unknown how this spectrum of musical engagement impacts development, social bonding, and other processes. Much of the research on the impacts of musical expertise has investigated how training affects relatively low-level communicative processes, such as early neural responses to simple speech sounds. For example, in two longitudinal studies, preschoolers showed enhanced auditory brainstem responses to speech in noise after 1 year of musical training (Strait et al., 2013), and adolescents in a school band showed a level of subcortical response consistency to syllables that was less degraded over the course of adolescence than in peers in a non-musical control group (Tierney et al., 2015). Some studies have found a modest benefit of musical training on speech segmentation (François et al., 2013) and phonological awareness (Degé and Schwarzer, 2011; Patscheke et al., 2016). Given that musical engagement early in life (e.g., singing or playing in a school choir or band) is often a highly rewarding and socially enriching experience, surprisingly little is known about the impact of early musical training on social processes, but formal music training has been associated with a decrease in relationship conflict in undergraduate students (MacDonald and Wilbiks, 2021).

Around the world, music plays a crucial role in important moments in life, from celebrations (festivals, weddings, graduations, inaugurations) to funerals. The extent to which collective music-making exists as an important part of social bonding is surprisingly variable across small-scale societies (Patel and von Rueden, 2021). In addition, there is significant variability across kinds of collective music-making in terms of whether most people (versus merely professionals) feel welcome to participate. For example, at weekly religious services, many congregants sing along regardless of vocal training (this is also true at karaoke bars and many sporting events around the world), but at other kinds of social occasions (dinner parties, talent shows) non-musicians often feel shy and ill-equipped to “perform” at a certain level. This variability in people’s engagement with various musical interactions also likely differs across cultures in ways that would be productive to study within a multi-brain framework.

While previous work has established musician/non-musician differences in processing communicative information (typically, simple music and speech sounds), future work must take into account this spectrum of everyday musical engagement and how it impacts music’s communicative functions throughout development and society at large. For example, it would be informative to use dual-brain hyperscanning paradigms to examine the difference between patterns of neural synchrony within pairs of highly trained musicians versus less trained musicians, or between pairs of people from a culture where music is more versus less widely practiced. In addition, studying differences in both musical and verbal communication between pairs of professional musicians who are accustomed to playing with each other versus strangers could help elucidate the neural overlap between these two domains of communication in more naturalistic, interactive contexts. For example, highly skilled jazz musicians who regularly “trade fours” are uniquely adept at matching certain aspects of other performers’ acoustic patterns to create coherent and complementary melodic lines; does this powerful “conversational” ability extend in any way to verbal communication [beyond body movement; (Robledo et al., 2021)] among such players, and do similar neural substrates support both of these processes?

## Music therapy can improve interpersonal connection and communication in clinical settings

One of the most powerful clinical applications of music is to improve communication among populations that struggle with verbal communication, self-expression, and/or social engagement. In children with autism spectrum disorder (ASD), music therapy has been shown to improve emotional and interpersonal responsiveness during the course of a session (Kim et al., 2009) and improve communication over an 8–12 week intervention (Sharda et al., 2018). Music has also been used in group settings to specifically improve communication and interpersonal relationships: group music therapy has been shown to improve social skills of children with ASD (Blythe, 2014) and family-centered music therapy has been shown to improve interpersonal relationships at home (Thompson, 2012; Mayer-Benarous et al., 2021 for a review).

Music therapy is also used to help improve communication for people with dementia. Music therapy strengthens interpersonal relationships between the patient and their music therapist, peers, and family (McDermott et al., 2014). People with dementia show more communicative behavior during a music therapy session (Schall et al., 2014) and an increase in language use and fluency after a session compared to a conversational therapy session (Brotons and Koger, 2000). Since listening to familiar music enhances self-awareness (Arroyo-Anlló et al., 2013) and autobiographical memory (El Haj et al., 2012a,b), for people with dementia, music may provide a means of connecting with the people around them and their past selves, thus helping them maintain a sense of personal identity as the disease progresses.

These studies document some of the cognitive and behavioral benefits of music therapy on communication, but there is more work to be done to fully understand these benefits and the neural mechanisms that give rise to them. A traditional approach would be to use neuroimaging pre- and post-intervention in an attempt to measure neural correlates of observed cognitive, behavioral, or social effects. Beyond that, hyperscanning approaches (e.g., with EEG or fNIRS) will be transformative in capturing the dynamic interactions between patients and music therapists or parents as they unfold in real time. For example, dyads of parents and non-verbal children become neurally synchronized during a music therapy session even when the parents are not active participants (Samadani et al., 2021). This could be evidence of cognitive-emotional coupling mediated by therapy and might be involved in strengthening the parent/child relationship. Isolating a neural signature or biomarker of communication success will provide useful feedback to improve the efficiency of not only music therapy but music education as well.

## Concluding remarks: future work would benefit from using music as a model for communication and taking into account complex brain-behavior dynamics

Because music is universal, has features that overlap and complement those found in speech, and plays a unique role in human emotional, social, and cultural experiences, it offers an ideal model for future studies on human communication. In this review, we have proposed that naturalistic, interactive music paradigms provide a rich opportunity to study the interpersonal dynamics of communication, and that communication research in general would benefit from a closer examination into the phenomenon of joint music-making (see Figure 1).

While previous studies have examined some aspects of musical communication, often focusing on the overlap between the processing of music and language at the acoustic or syntactic level, most paradigms use fairly simplistic stimuli and individual participants (rather than dyads or groups) or have focused on inter-brain mirrored synchrony rather than modeling the complex dynamics inherent in real-life musical interaction. Further, the existing literature has largely ignored the influence of musical experience on communication as well as the power of music to transfer emotions between people. Future studies must take into account the dynamic nature of communication and the parallel features that must be coordinated across performers (e.g., timing, tuning, leader/follower dynamics), going beyond the single- or even dual-brain models that have previously been used. Naturalistic paradigms featuring real-life musical interactions and stimuli are best suited to address these complex questions and relationships, and findings have the potential to inform avenues for future therapeutic interventions.

## Author contributions

SI and EP contributed to the conception of the topic. All authors wrote the sections, contributed to revision, and read and approved the final manuscript.

## Conflict of interest

The authors declare that the research was conducted in the absence of any commercial or financial relationships that could be construed as a potential conflict of interest.

## Publisher’s note

All claims expressed in this article are solely those of the authors and do not necessarily represent those of their affiliated organizations, or those of the publisher, the editors and the reviewers. Any product that may be evaluated in this article, or claim that may be made by its manufacturer, is not guaranteed or endorsed by the publisher.

## Supplementary material

Supplementary Video 1 Demonstration of the dynamic joint actions required to support successful musical communication in a similar small group, a woodwind quintet.
